# Transition metal attenuated mechanism for protective alumina formation from first principles†

**DOI:** 10.1039/c8ra08195f

**Published:** 2018-12-11

**Authors:** Vedad Babic, Christine Geers, Itai Panas

**Affiliations:** Department of Chemistry and Chemical Engineering, Chalmers University of Technology Gothenburg 41296 Sweden vedadb@chalmers.se

## Abstract

A mechanistic perspective on the growth of protective oxides on high temperature alloys at elevated temperatures is provided. Early, defect rich transient alumina is understood to form by outwards diffusion of oxygen vacancies and electrons. The impact of transition metal (TM) ions (Sc, Ti, V, Cr, Mn, Fe, Co, Ni) on the oxygen vacancy diffusion and electron transport in α-alumina was studied by employing density functional theory. Activation energies for electron transfer *E*_A_(ET) between oxygen vacancies in pure as well as TM doped α-alumina were subject to analysis, and similarly so for the TM and charge dependent activation energy for oxygen vacancy diffusion *E*_A_(*V*_O_). *E*_A_^*Q*^(ET) were found to be ∼0.5 eV while 2 eV < *E*_A_^*Q*^(*V*_O_) < 5 eV was obtained. The higher and lower *E*_A_^*Q*^(*V*_O_) values correspond to uncharged and doubly charged *V*_O_ sites, respectively. Redox processes among *V*_O_ sites, addressed by a bipolaron approach, were understood to enhance *V*_O_ mobility and thus to facilitate oxide growth. TM adatoms induced asymmetry in the potential energy surface for oxygen vacancy diffusion was subject to analysis. Competition for electrons between all-Al^3+^surrounded oxygen vacancies and vacancies adjacent to the late 3d adatoms comes out in favor of the latter. A novel take on the 3rd element effect in FeCrAl emerges from analysis of the ternary TM–TM*–Al system.

## Abstract 2

A mechanistic perspective on the growth of protective oxides on high temperature alloys at elevated temperatures is provided.

## Graphical abstract

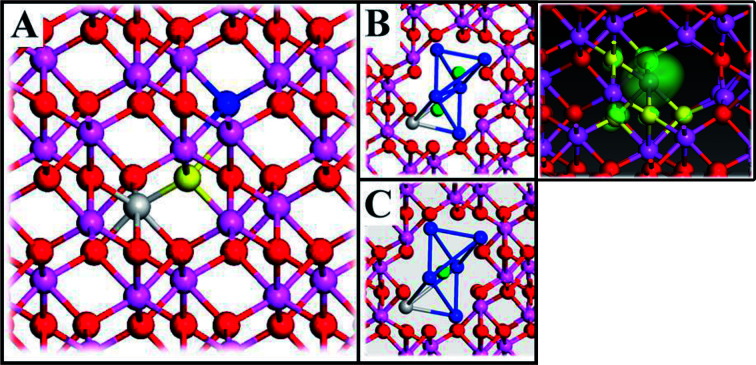

## I. Introduction

In high-temperature applications, stainless steels and FeCrAl alloys are used owing to their characteristic ability to grow dense and adherent oxide scales, *i.e.* composed of chromia or alumina, in order to protect the alloy from the corrosive environment. The target for these optimized slow-growing scales is that the alloy becomes limited only by the critical creep levels of the base metal.^1^ Ways to enhance alloy resilience include addition of oxide scale forming components as well as enhancing additives for improved scale adherence.^2^

The conceptual approach commonly resorted to in alloy oxidation studies employs non-equilibrium thermodynamics. Here the oxidation process is taken to reflect an ongoing transformation of oxidizing and reducing agents, commonly O_2_ and the alloy itself, that are continuously converted into new oxide scale. The oxidation is understood to be driven by a chemical potential difference across the resulting scale and is sustained by Fickian diffusion processes that result in parabolic or sub-parabolic scale growth kinetics. In a Wagnerian setting, this is realized by charged mobile entities; ions, vacancies, and electrons. Hence, the oxidation is sustained owing to electron transfer across the barrier oxide whereby new anions and cations are produced, here O^2−^ and Al^3+^, respectively. Correspondingly, anionic and cationic vacancies are formed at the anode and cathode. Thus, scale growth is maintained by the continuous formation and annihilation of said excess ions by the corresponding vacancies.

The purpose of the present study is to shed light on central mechanisms involved in oxidation processes supporting the protective oxide scale formation on alumina forming alloys. Although this topic has been the subject of extensive investigations in the past, the transport properties of the alumina scale are still under debate.^3–6^ In particular defect and vacancy related properties have been studied extensively, both by means of experiment and theory, extracting mainly ions migration and formation energies in pure as well as doped alumina scales.

Here we underline the crucial impact of the transient phases during scale formation and growth from an initial rapidly forming mixed “junk oxide”. Consequences of initial stages of high temperature oxidation include the subdivision of the resulting oxide scale into outer equiaxial and inner columnar grains.^5^ The outer defect rich gamma alumina and the inner barrier oxide, composed of slow-growing α-alumina are separated by a thin chromia component commonly understood to reflect the position of the initial alloy surface.

Often, low concentrations of reactive elements, RE (Zr, Hf, Y) are added to the alloy to improve on corrosion resistance by enhancing the adherence of the thermally grown scale. The RE do not dissolve in the oxide lattice. Instead, these ions segregate into the grain-boundaries, where most of the ionic transport occurs. In this context, a beneficial transient rapidly growing “messy” alumina was recently described,^8^ where the cause for the initial accelerated oxidation was the cooperative effect of water and yttria. Along this line, oxygen diffusivities in RE doped scales are several orders of magnitude greater than the Al^3+^ diffusivities^9^ despite the fact that activation barriers for the two have been reported to be of similar size (∼6 eV).^10^ Indeed, the discrepancy between measured and calculated values for oxygen vacancy activation barriers has been coined the “corundum conundrum” that is partly owing to shortcomings in employing sintered alumina to model thermally grown alumina.^11^ It is conceivable that due to the transient nature of the thermally grown oxide, its grain-boundaries and short-circuit diffusion paths come out different from those of sintered alumina, physically as well as chemically. While thermally growing oxide scales are formed in an electrochemical potential gradient and away from equilibrium, activation energy measurements are often performed on single or polycrystalline alumina which are spontaneously formed^12^ or sintered.^13^ Thus, measured activation barriers need not necessarily be directly applicable to alloy oxidation conditions.

The thermodynamic drivers and the subdivision of time scales for the various processes offer essential clues for understanding barrier oxide growth on high-temperature alloys. Thus, stabilities of different phases in the oxide scale – the grains being subject to Ostwald ripening – are taken to reflect the local equilibrium conditions as determined by fictitious local oxygen partial pressures ranging from *p*O_2_ at the gas/oxide interface to the dissociation pressure of the oxide at the alloy/oxide interface. Achieving the latter is taken to be the slowest process associated with oxide scale growth. Intermediate is the transformation from the mentioned initial transient oxide to the one that satisfies said local equilibrium conditions.^7,14–22^

The properties of the transient oxide are indeed crucial for the alumina forming process, partial control of which may be gained by the so-called 3rd element effect. As first observed in the Cu–Zn–Al system,^23^ the 3rd element effect is understood to initiate and maintain formation of a protective α-Al_2_O_3_ scale. In FeAl alloys, internal oxidation is avoided by a sufficient chromium addition whereby outer oxidation is achieved, leaving a remnant outer iron oxide on top of a transient mixed (Al,Cr)_2_O_3_ scale.^7,14,19^ This scale in turn seeds α-Al_2_O_3_ formation. Explanations of the 3rd element effect evolve around suppression of the oxygen activity at the alloy/oxide interface.^15,22^ This oxygen deficiency perspective lends additional relevance to the present study which explores the impacts of adatoms on *V*_O_ mobility and *V*_O_ mediated electron conduction.

In the sub-parabolic slow late stages of scale growth, α-alumina comprises an inward-growing oxide as oxygen vacancy transport becomes the rate-limiting process. However, inasmuch as α-alumina is a large band-gap insulator, the conduction band becomes inaccessible for electron transport. Here, we explore possible aspects of electron transport that utilizes oxygen vacancies to acquire mobility. Inspiration is partly drawn from recent calculations highlighting the impact of oxygen vacancy charge on the activation energy for oxygen vacancy diffusion, *i.e.* ∼5 eV for neutral oxygen vacancies (*V*^x^_O_), and ∼2 eV for doubly charged vacancies 
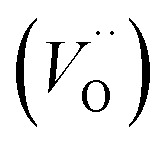
.^24–26^ The latter study was inspired by experiments on thin alumina insertions in resistive-switching random access memories.^27^While electron and vacancy mobility must be a correlated process, the two entities do not need to move as one unit. Instead, we understand the existence of charged anion vacancies at steady-state to offer percolating electron transport channels from the anode to the cathode, resulting in the quasi-independent motion of electrons and oppositely charged oxygen vacancies, the latter providing electronic impurity states in the alumina band gap, see 
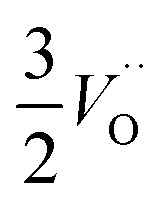
 in (R2) and (R3), above.R1
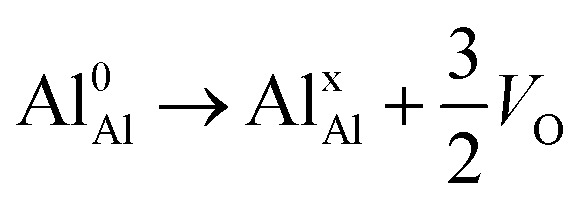
R2
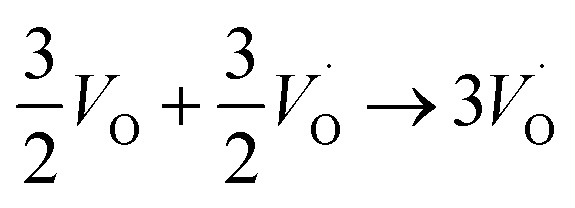
R3
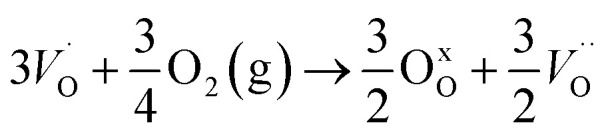


Moreover, in as much as polarons comprise electrons sitting in potential wells (traps) created by the lattice distortions, polaronic transport mechanisms are employed to describe how electrons migrate from site to site *via* a thermally activated hopping mechanism (adiabatic) or quantum tunneling (non-adiabatic). Electron transport in oxides employing such a polaronic transport mechanism has been reviewed by among others Holstein,^28,29^ Marcus,^30^ Emin,^31^ and Alexandrov and Mott.^32^ This theoretical framework has been applied to among others α-chromia,^33^ hematite,^34^ TiO_2_ (ref. 35) in which cations are taken as polaron trapping sites, and *m*-HfO_2_ (ref. 36) where oxygen vacancies are taken as polaron sites. In the present work we employ the thermally activated hopping approach developed by Marcus,^30^ where either 
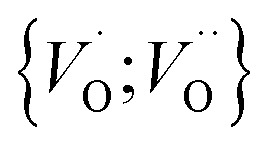
 site occupations become converted into 
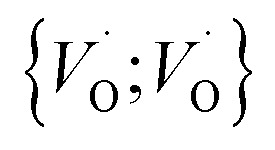
, 
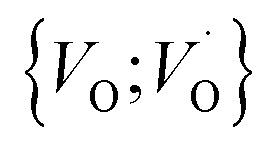
 into 
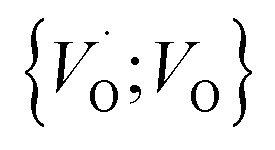
 or 
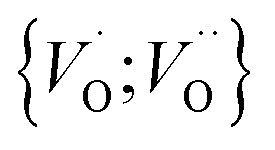
 into 
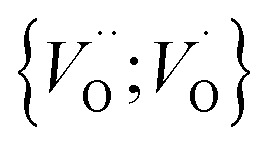
 owing to the corresponding activated electron transfers. In what follows, the concept of cooperative mobility of electron and charged oxygen vacancy is validated systematically by utilizing first-principle density functional theory calculations. Moreover, support is provided for the notion that percolating electron conduction assigns variable charges to the oxygen vacancies. Bearing in mind that the mobility of the latter increase with their charge, the activated electron hopping is said to facilitate the complementary mobility of oxygen ions.

In what follows, we assume the polaronic electron mobility and the oxygen vacancy diffusion to be much faster than the rate of transient oxide conversion into a scale that obeys local equilibrium conditions. The impact of TM {Sc, Ti, V, Cr, Mn, Fe, Co, Ni} on adjacent *V*_O_, 
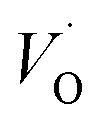
 and 
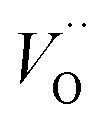
 is considered in order to expose generic features and formulate descriptors extracted from the local electronic and lattice structures. Thus, charge dependent oxygen vacancy mobility is considered in III.A. Characteristics of five different electron hopping processes between oxygen vacancies in the high and low TM doping limits are discussed in III.B, while potential implications from our findings as to the third element effect are discussed in III.C.

## II. Modelling considerations

### II.A. Computational details

The Density Functional Theory (DFT) calculations in this article were performed using the CASTEP^37^ code within the Materials studio 6.0 suite.^38^ The PBE-GGA functional^39^ was employed for all calculations. Non-local Vanderbilt ultrasoft pseudopotentials^40^ have been used to describe core electrons together with an energy cut-off *E*_cut_ = 400 eV and the Brillouin zone was sampled on a 2 × 2 × 2 Monkhorst–Pack grid.^41^ The convergence criteria included 10^−3^ Å (displacement) and 10^−5^ eV per atom (energy). The transition state (TS) searches were performed using a complete LST/QST search protocol^42^ and an RMS convergence of 0.05 eV Å^−1^. Spin-polarized calculations were performed throughout.

**Fig. 1:**
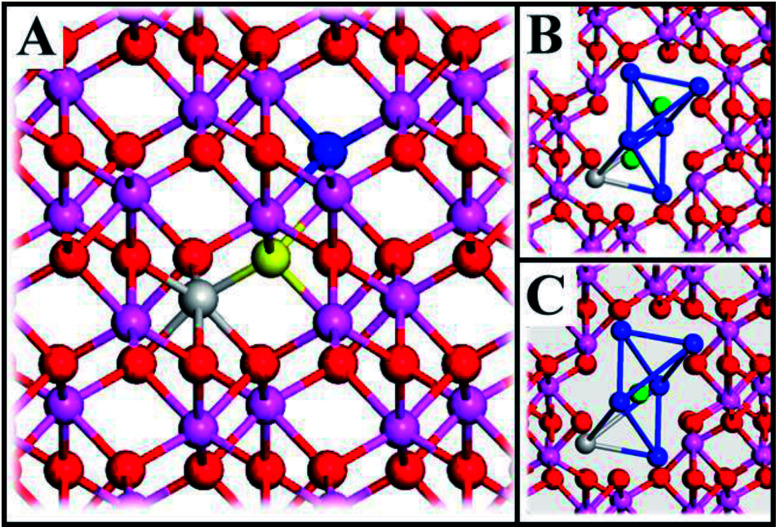
(A) Atomic structure of alumina (Al – purple, O – red, and blue and yellow for oxygen which are removed to form a *V*_O_, including one TM^3+^ (gray)). The initial *V*_O_ position (“Start”) is formed by deleting the yellow O-atom and the final position (“End”) by deleting the blue O-atom. (B) and (C) show the eight cations (blue – Al, gray – Al, TM) which comprise the two distorted tetrahedra whose volumes are referred to as vacancy volumes. In (B) two O^2−^ ions (greens) represent the ions that are removed to form “Start” and “End” structures, respectively. In (C) the transition state of the oxygen vacancy diffusion process is visualized.

PBE0 and GGA-PBE calculations have been compared by single-point calculations of the three relaxed structures, see Fig. 1 employing the previously mentioned parameters. Here, norm-conserving pseudopotentials with an energy cut-off of 600 eV have been used in conjunction with a gamma point (1 × 1 × 1) Monkhorst–Pack grid. Convergence criteria include 2 × 10^−6^ eV per atom (energy). The band-gap was calculated on a primitive cell of alumina with a density-of-states (DOS) calculation employing 0.02 Å^−1^*k*-point sampling, all other parameters remained unchanged.

### II.B. Structural model

The system consists of a 2 × 2 × 1 supercell (9.518 × 9.518 × 12.991 Å^3^) of α-Al_2_O_3_ (rhombohedral *R*3*c*) with one Al-atom substituted for a TM (Sc, Ti, V, Cr, Mn, Fe, Co, Ni) and an oxygen vacancy which is formed by deleting one O-atom, see Fig. 1A. When vacancy volumes are referred to, they refer to the sum of volumes of two tetrahedra comprising of seven Al cations (blue) and one cation (Al, TM; grey), see Fig. 1B and C (green ions represent O^2−^ ions equivalent to the yellow and blue O^2−^ in Fig. 1A). The resulting model consists of 71 O-atoms, 47 Al-atoms and one TM and is either neutral or charged (*Q* = 0, +1, +2). Here, we identify properties obtained from straight-forward systematic calculations involving Sc, Ti, V, Cr, Mn, Fe, Co, Ni substitutional doping at Al^3+^ sites of α-Al_2_O_3_ bulk.

### II.C. Modelling transport

While oxide scale growth is the result of the interconnected transport of oxygen and electrons, the theoretical approaches to learn of their mobility differs. Oxygen diffusion is treated by simple transition state (TS) theory where the transition state for oxygen vacancy diffusion is found by employing a complete LST/QST scheme. The “Start”, TS, and “End” geometries are analyzed by referring to the corresponding electronic structures as described by their partial density of states (PDOS). Moreover, charge and dopant dependent activation energies for displacement of the vacancy are extracted.

**Fig. 2:**
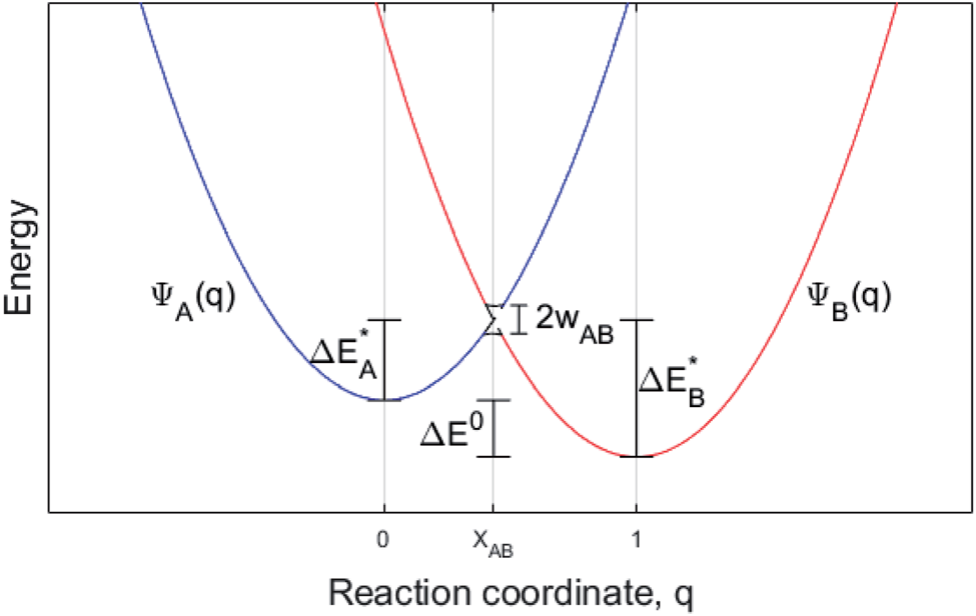
Potential energy curve *vs.* reaction coordinate for a polaron transport process where an electron transfers from site P_1_ to P_2_. B_A_ is then the initial configuration; (P^red^_1_;P^ox^_2_), and B_B_ is the configuration after the electron transfer, *i.e.* (P^ox^_A_;P^red^_B_). *ψ*_A_(*q*) and *ψ*_B_(*q*) denote the potential energy curves for B_A_ and B_B_, respectively, and *X*_AB_ the crossing between the two along the reaction coordinate *q*. 
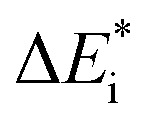
 is the activation energy of the electron transfer between configurations i = A, B, and Δ*E*^0^ refers to relative stability of B_A_ and B_B_. The electronic coupling constant, *w*_AB_, is neglected in the present study.

The electron transport study relies on small polaron hopping theory which is analogous to the electron transfer framework developed by Marcus.^30^ Here we employ the terminology used in Marcus theory. Relevant parameters become activation energy 
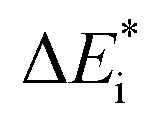
, electronic coupling constant *w*_AB_, and reaction energy Δ*E*^0^, see Fig. 2. The potential energy curves *ψ*_A_(*q*) and *ψ*_B_(*q*) reflect the electron transfer process between sites 1 and 2. Polarons, P are ascribed to each of the sites, such that the electron transfer process becomes bipolaronic in nature. The bipolarons B_A_ and B_B_ refer to *ψ*_A_(*q* = 0) and *ψ*_B_(*q* = 1), respectively, such that the electron transfer process may be expressed asB_A_(P^red^_1_;P^ox^_2_) → B_B_(P^ox^_1_;P^red^_2_)

The transfer may only take place when the two curves cross at *q* = *X*_AB_; *ψ*_A_(*X*_AB_) = *ψ*_B_(*X*_AB_). In the present study, two well separated oxygen vacancies are understood to accommodate the bipolarons, their structures reflecting the corresponding charge state, *i.e.* whether reduced or oxidized. Note that the bipolaron description B_A_(P^red^_1_;P^ox^_2_) employed here does not emerge from a single calculation but requires treating P^red^_1_ and P^ox^_2_ separately. The reason is that LDA and GGA are unreliable in describing localized electrons, *e.g.* for a singly charged super cell (*Q* = +1) two equivalent polaron sites may render the electron delocalized due to the self-interaction error SIE such that each site takes on *Q* = +1/2. Also for inequivalent polaron sites, potentially erroneous partial charges may emerge owing to the SIE. This is remedied here by the coupling of super cells, each containing a single polaron site that is described separately. Thus, per default a discrete number of electrons reside in each polaron site. In effect, the bipolaron is thus represented by two small polarons, one representing the reduced donor site and one the oxidized acceptor site, and where the small polaron self-energies of P^red^_1_ and P^ox^_2_ are realized by the separate super cell calculations. The transfer between two distant vacancies is conditioned by the total energy of P^red^_1_ + P^ox^_2_ being equal to P^ox^_1_ and P^red^_2_ as achieved by coupled linear deformations, see Fig. 2. In addition, the model requires that the overlap between electronic states at P_1_ and P_2_ is negligible, also consistent with the definition of small polarons.

The emerging activation energy for electron transfer, 
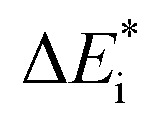
, is calculated together with the reaction energy Δ*E*^0^ assuming that *w*_AB_ is zero. Thus, an upper bound for 
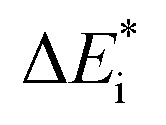
 is arrived at. Below, the assumption that the oxygen vacancy diffusion is rate-limiting is validated, in support of the notion that during alloy oxidation electron transport in nominal large band-gap insulators utilizes oxygen vacancies for easy percolating electron transport.

### II.D. Basis of fundamental assumptions

Several implications of our work rely on the exponential near-sightedness (ENS) of orbital interactions in ionic large band gap insulators. The ENS emphasizes the importance of local inter-atomic orbital overlaps and local coulombic interactions to determine the essential transport properties rather than any long-range interactions *e.g.* coulombic, or non-local quasi-free particle descriptions. The long-range contributions to the coulombic interactions are slowly varying in space and the differential effect on the length scale (typically a few Ångström) of a local diffusion process is expected to cancel. As for explicit non-local wave function properties, neither the metallic free-particle description nor any entanglement driven non-locality is deemed relevant here. This is in contrast to *e.g.* formation energy calculations which rely on energy comparisons of perfect and defect containing crystals *e.g.* bulk alumina and alumina containing a charged vacancy.^43,44^ Repeatedly, it is noted that upon charging a periodic super cell, the total energy calculation diverges due to an infinitely periodic array of charged cells. This is remedied by removing the monopole contribution to the total energy, which is physically equivalent to introducing a compensating homogenous background charge.^45^ Here, stability differences are computed, and care is taken to make model dependent artefacts cancel.

The near-sightedness has the “End” position to be energetically equivalent to any other analogous oxygen vacancy position *x*, which is not a nearest-neighbor NN to the TM-substituted Al-position *i.e.* E[“End”] = E[*x*], *x* ≠ NN. The degree of validity of this assumption is tested by calculating the energies for various *x*'s. Thus, stabilities for Sc-, Ti-, and Co-doped alumina with vacancies formed at a TM–*V*_O_ distance of 2.0 Å (NN = “Start”) are compared to structures for *x* ≠ NN, *i.e.* 3.5 Å, 3.9 Å, 4.4 Å (“End” position), employing the stability of the vacancy position at 5.1 Å as reference, are provided in Table S1.†

The nominal vacancy charge state (*Q* = 0, +1, +2) is often referred to, while in practice it is only possible to give meaning to the charge state of the super cell. Often, the charge state of the vacancy is indeed the same as the charge state of the super cell, *e.g.* in absence of redox active species. However, upon doping alumina with transition metals, some redox active, this assumption is no longer valid. This is addressed in more detail below.

## III. Redox assisted oxygen vacancy mobility in transient alumina

In what follows, an electron transport controlled and TM attenuated, mechanism for enhancing the mobility of oxygen vacancies is unraveled and articulated. It is decisive for the diffusivity of oxygen ions and thus the rate of oxide growth. Dual consequences emerge of the stability of the transient oxide, as assessed by the affinity of oxygen vacancies to TM ions and taken to delay the transformation process. On one hand the ‘junk oxide’ grows thicker, while on the other hand it displays better adherence to the alloy support at the crucial early stages of scale formation preceding the slow-growing barrier oxide at late stages.^8,46^

### III.A. Transition metal attenuated oxygen vacancy mobility

**Fig. 3:**
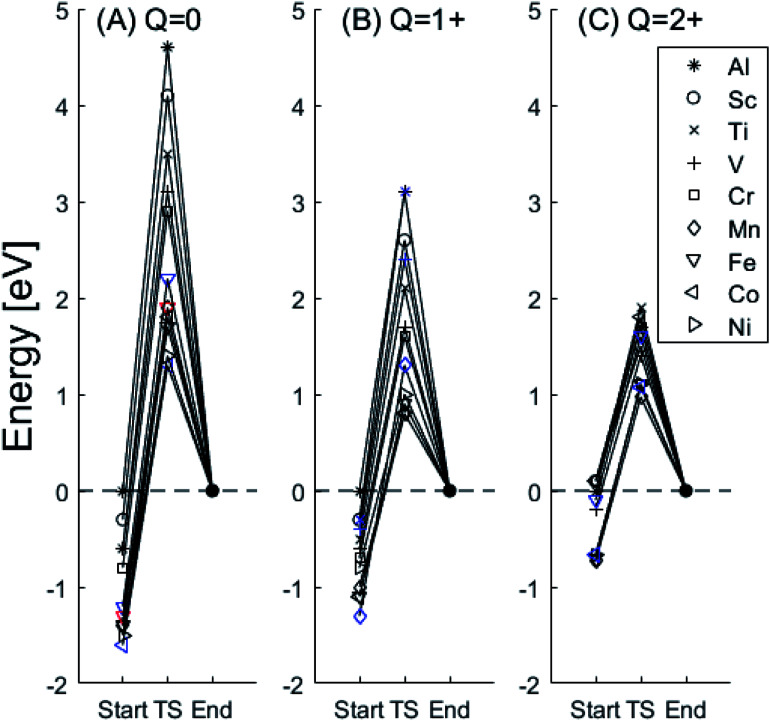
Potential energy surface along the diffusion path for (A) *Q* = 0, (B) *Q* = +1, and (C) *Q* = +2, with transition metal ion at the origin. The “Start”, transition state (TS) and “End” structures are indicated (compare Fig. 1). (A) Pinning of *V*_O_ at “Start” is seen to increase dramatically with increased atom number. This is in contrast to (C) 
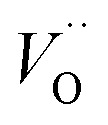
 where pinning is negligible except in some special cases where a redox reaction occurs when charging the supercell and thus the vacancy is +1, with TM^2+^. Also, the activation energy on passing the TS decreases on *V*_O_ approaching the TM^3+^ site (A). This is again in contrast to 
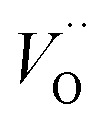
. (B) comes out in the middle of (A) and (C).

**Fig. 4:**
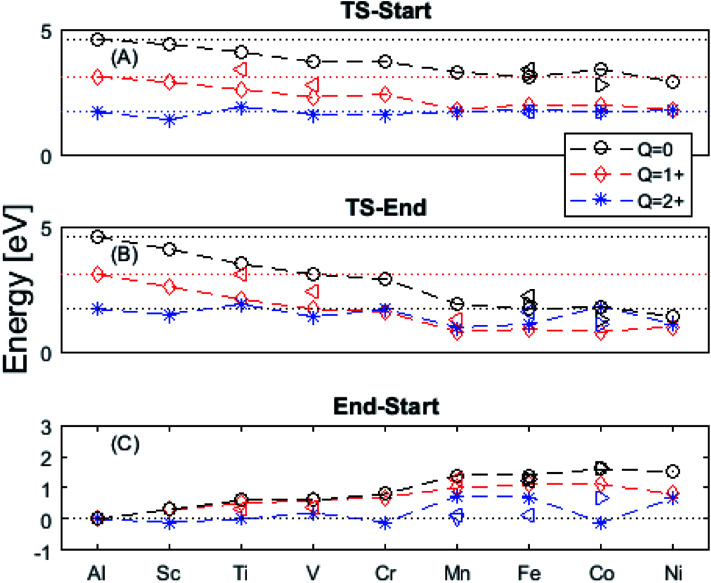
Activation energies from (A) “Start” and (B) “End” structures as well as (C) the pinning energy, “End”–“Start”. Triangles denote exited spin states. Observe that *Q* = +2 often shows the same behavior as undoped alumina (dotted line) regardless of doping. Mn, Fe, and Ni ground states deviate from this behavior, though excited spin-states have been found which obey the earlier order. An excited Co spin-state has been found in which a pinning effect is observed for *Q* = +2.

In the following we explore the impacts of two TM adatom associated properties that, together with the charge, jointly become decisive for the mobility of oxygen vacancies. One property concerns the activation energy for diffusion and the second relates to the asymmetry of the potential energy surface APES. Thus, calculations tell of enhanced APES for neutral vacancies. Preference to reside at a site adjacent to the TM ion is found, and increasingly so with increasing atomic number, see Fig. 3 and 4 and Tables S2–S4† for numerical values. The APES is due to compound formation between TM and *V*_O_, *vide infra*, while symmetry is recovered with increased nominal charge *Q* of the oxygen vacancy, *i.e.* APES(0) > APES(+1) > APES(+2). The APES is accentuated in particular for ground states of Mn, Fe, and Ni, while it is small in case of Co. The latter may be changed by considering an excited spin state of Co. Indeed, APES(+2) was observed to appear or disappear also in case of Mn and Fe, depending on the particular electronic state of the adatom. This effect is due to orientation of valence orbitals, whether along or perpendicular to the O–TM–*V*_O_ axis. In case of the orbitals pointing along this axis, geometrical relaxations cause the O–TM distance to increase due to Pauli repulsion, which in turn causes the APES, see Section S1† for details.

**Fig. 5:**
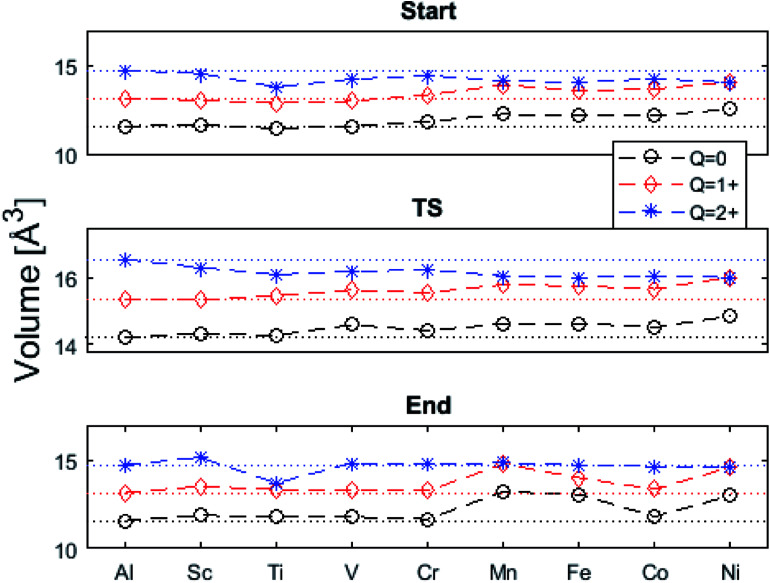
Vacancy volumes at the different locations. “Start” and TS show similar behavior where *Q* = 0, +1 have an increase in volume for Mn, Fe, Co, and Ni while *Q* = +2 seems to decrease with atomic number. “End” state, being physically removed from the TM tests the vacancy charge. Ti(*Q* = +2) shows the Ti to donate an electron to the vacancy, which takes on *a* +1 charge state. Mn, Fe, and Ni are reduced in *Q* = 0, +1.

**Fig. 6:**
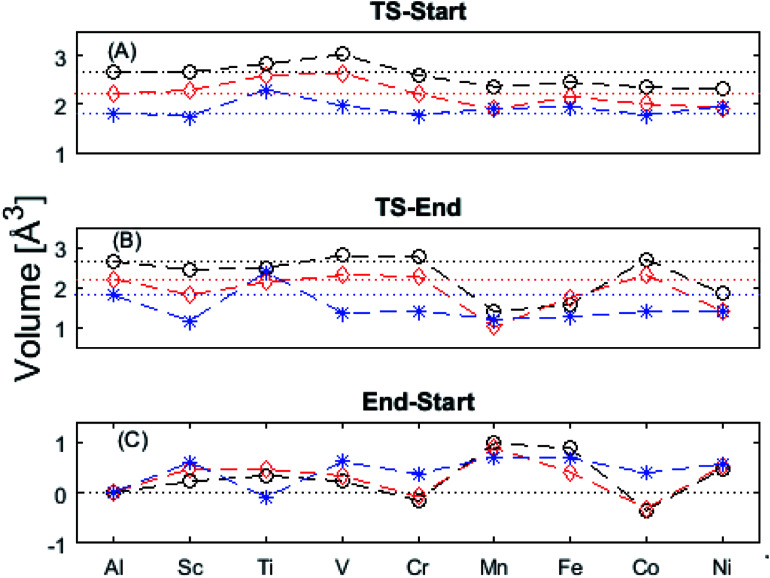
Vacancy volume differences at “Start”, transition state (TS), and “End”. Similar to Fig. 4(A) and (B), in both (A), and (B) *Q* = 0, and +1 follow each other with an offset and merge with *Q* = +2 for Mn, Fe, and Ni. TS and “Start” show similar behaviors reflecting gradual electron polarizability associated volume changes with increased atomic number, while the “End” state, owing to the large geometric distance to the TM ion, is either reduced, oxidized or unchanged. This results in the drastic volume changes in (B) and (C).

The underlying cause for the increase in APES with atomic number is explored further. Consider the changes in sum of volumes of the two distorted tetrahedra comprising “Start” (3Al & TM) and “End” (4Al) coordination of the corresponding oxygen sites, see Fig. 1B and C. Dependences of volumes on charge of oxygen vacancy (*Q* = 0, +1, +2) along the reaction coordinates (at “Start”, TS, “End”) are plotted for each of the adatoms, see Fig. 5 and 6, where horizontal lines refer to *V*_O_^*Q*^ of pure alumina. The sum of oxygen site volumes in “Start”, TS, and “End” are shown in Fig. 5. It is noted that TS and “Start” volumes are similar in exhibiting little structural information, and are understood to have similar physical origin. This is also consistent with Fig. 4A, where the TS-“Start” energies (*Q* = 0, +1) decrease with atomic number while *Q* = +2 remains more or less constant, similar to the TS-“Start” activation energies (Fig. 4). In case of *Q* = 0, +1 this is due to increasing stability of TM–*V*_O_ compound with increasing atomic number. As *V*_O_ in the “End” structure is disconnected from the TM ion, unlike “Start” and TS, the corresponding volume can be used to test the validity of the nominal charge state of the vacancy. Indeed, more distinct volume deviations from the nominal are observed in case of the “End” structures, taken to reflect the true charge of the oxygen vacancy. Hence, Mn, Fe and Ni nominally ascribed to take oxidation state +III, undergo redox in the presence of nominal *Q* = 0, resulting effectively in TM(ii) and *Q* = +1. Similarly, for nominal *Q* = +1 the TM(ii) oxidation state of Mn, Fe and Ni enforces the resulting effective *Q* = +2. Only for nominal *Q* = +2 do Mn, Fe and Ni remain in the assigned +III oxidation state. It is gratifying to note that Ti displays the complementary behavior, *i.e.* maintains +III oxidation state for nominal *Q* = 0 and *Q* = +1, while for nominal *Q* = +2, Ti accesses the +IV oxidation state enforcing the resulting *Q* = +1 charge of the oxygen vacancy. And this while Al, Sc, V, Cr and Co all come out +III for all three values of *Q*. Hence, the large variation in activation energies for oxygen vacancy diffusion in case of nominal *Q* = 0, *i.e.*Fig. 3A is partly owing to that Mn, Fe, and Ni should belong to *Q* = +1, *i.e.*Fig. 3B. Similarly, Mn, Fe, and Ni data for nominal *Q* = +1 should belong to *Q* = +2, *i.e.*Fig. 3C.

A further consistency check for the emerging understanding of the variations related to the “End” structures, with crucial impact on the TS, includes comparisons between ground states and excited states. Thus, by considering suitable excited states the charge transfer states can be made to enforce the nominal +III charge for Mn, Fe, and Ni in conjunction with *Q* = 0 for the oxygen vacancy. Analogously, by exciting the *Q* = 0 state in case of Co(iii) charge transfer into *Q* = +1 and Co(ii) can be obtained. In as much as the activation energy for diffusion is sensitive to the resulting charge of the oxygen vacancy, *Q* = 0, +1, +2 for Al(iii) in Fig. 3, the ambiguity of the TM effect is partly resolved.

**Fig. 7:**
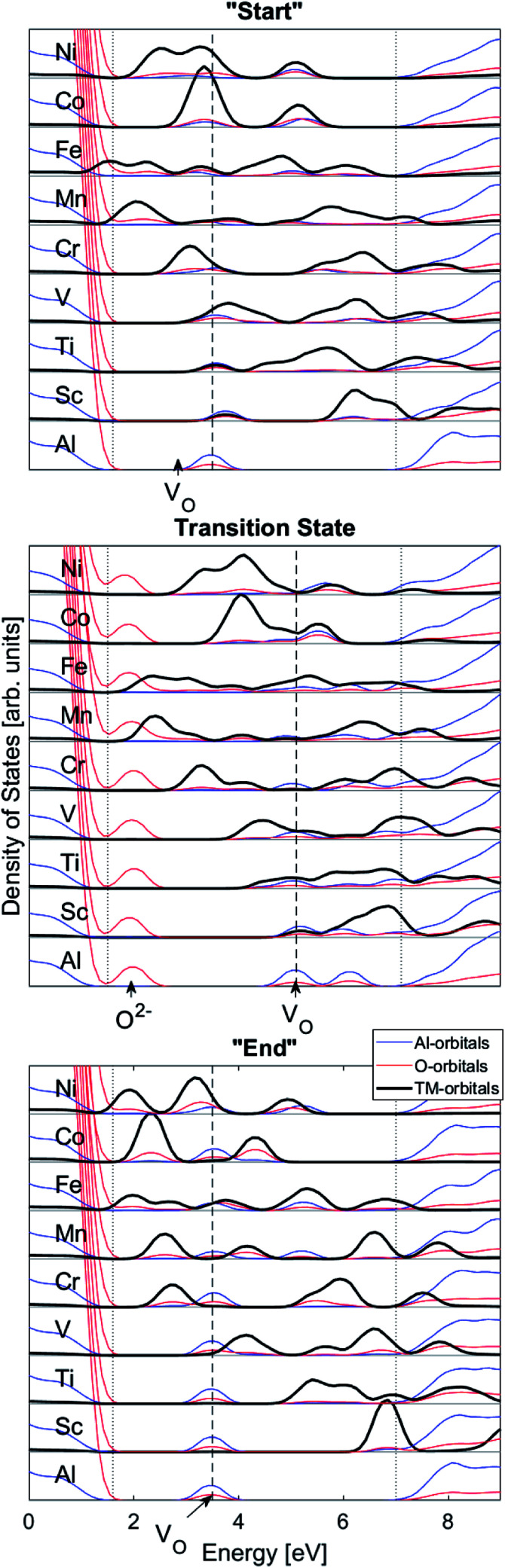
Total density of states (DOS) subdivided into transition metal (TM), Al, and O partial density of states (PDOS). “Start”, transition state, “End”: TM bands are split and down-shifted with increasing atom number. “Start”; the oxygen vacancy *V*_O_ band overlaps with the TM states. Transition state; the TM–*V*_O_ overlap remains, and in addition an O^2−^ state comes out of the valence band. “End”; TM–*V*_O_ overlap disappears as seen in mismatch of TM and *V*_O_ band energies. Dotted lines emphasize positions of top of valence band and bottom of conduction band.

We may search for further clues as to the origin of the APES by inspecting the changes in electronic density of states (DOS) and partial DOS (PDOS) along the reaction coordinate, from the starting configuration where *V*_O_ is situated adjacent to TM ion, *via* the transition state (TS) to the “End” configuration where *V*_O_ is situated close to Al^3+^, see Fig. 7 and compare Fig. 1 again.

Several characteristic features emerge:

(1) It is observed how the band associated with *V*_O_ is shifted to higher energies at the transition state (TS) and that it comes down deeper upon binding to the TM (Fig. 3).

(2) Apparent broadening of the *V*_O_ band is caused by its “dissipation” by mixing with a subset of the TM 3d manifold. Corresponding PDOS is rebuilt in response to this *a priori* mutual spatial and energy overlaps that increase with higher atomic numbers.

(3) An O^2−^ state dissociates from the oxygen dominated valence band on moving *V*_O_ towards the transition state and it returns to said band at the end points of the transfer process.

(4) On increasing atomic number the 3d states are stabilized,

(5) The mixing of TM and *V*_O_ PDOS indicates compound formation between TM and the oxygen vacancy, reflecting enhanced stability at the “Start” and TS geometries (see Fig. 7a and b).

(6) The absence of mixing of TM and *V*_O_ PDOS between the oxygen vacancy states and TM in the “End” structure reflects the greater spatial separation between *V*_O_and that TM is partly responsible for the APES.

It is indeed gratifying to note the extent to which the activation energy for oxygen vacancy diffusion probes the electronic occupation of the associated *V*_O_ state. The activation energy for 
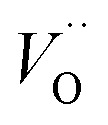
 upon passing the transition state corresponds to the out–in motion of the O^2−^ ion associated band being displaced simultaneously with the vacancy. This reflects that both the isolated O^2−^ band and the *V*_O_-associated bands become displaced to higher energies at the TS, thus explaining why 
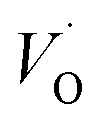
 (*Q* = +1) comes out intermediate to *V*_O_ (*Q* = 0) and 
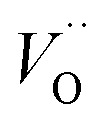
 (*Q* = +2), *cf.*Fig. 3 for potential energy curves. Indeed, in case of *V*_O_ (*Q* = 0) diffusion both the O^2−^ band and the *V*_O_ band are filled whereas only the O^2−^ band is occupied in case of 
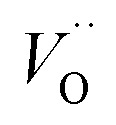
. This is consistent with other studies,^24^*i.e.* explaining the special case of pure alumina where the barrier for diffusion comes down from ∼5 eV for *V*_O_ to ∼2 eV for 
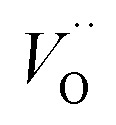
.

**Fig. 8:**
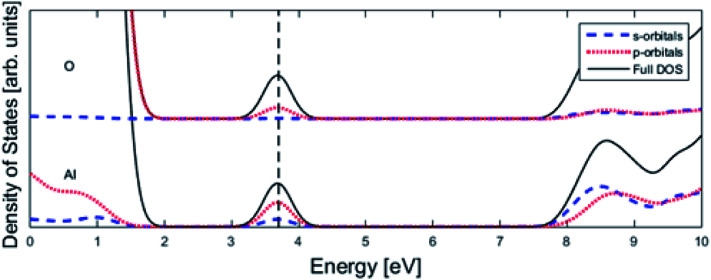
Total density of states (DOS) subdivided into Al partial density of states (PDOS) and O PDOS contributions for pure alumina with one oxygen vacancy (see Fig. 1), whose associated impurity band is found at the Fermi level (vertical dashed line). The main contribution is coming from p-orbitals.

Finally, the origin of the *V*_O_ (*Q* = 0) pinning to TM ion was subject to further analysis. First, in agreement with previous work, we observe that *V*_O_ is described by aluminium states. However, while the Al 3s orbitals were said to constitute the oxygen vacancy band,^47^ here PDOS results presented in Fig. 8, show the main contribution to be mainly from Al 3p-orbitals. In TM substituted alumina, the density of states in the band gap of alumina is dominated by the TM d-orbitals, see Fig. 7 for DOS figure, apparently showing also partial DOS contribution from TM 4s and 4p bands. In order to further extract the impact of the 3d shell on the shape of the potential energy landscape, the TM was replaced by Ga. Inasmuch as Ga displays a 3d^10^ filled sub-shell it becomes constrained to only offering 4s and 4p orbitals in the binding to *V*_O_. Validation of our understanding concerning the approximate spectator role of the 3d shell was indeed found in that the nominal Ga(iii) (*Q* = 0) APES is similar to that of nominal Co(iii) (*Q* = 0). However, the activation energy for diffusion comes out significantly higher for Ga(iii) (*Q* = 0) than that of Co(iii) (*Q* = 0), see Table S2.† Reflecting on this finding, it is gratifying to observe that the electrons occupying the *V*_O_ all-metallic state, besides utilizing the 3s and 3p orbitals on adjacent Al^3+^ ions also employ the analogous 4s and 4p orbitals of the transition metal ion. And while *V*_O_ bonding to the 3d shell affects only marginally the “valleys” of the potential energy landscape, its decisive impact owing to charge transfer between TM and oxygen vacancy at the transition states is repeatedly emphasized. It controls the activation energy for diffusion by allowing electron transfer from the *V*_O_ to the TM, *i.e.* between corresponding electronic states at the Fermi level (*E*_F_), which offers intersystem crossing at the transition state. Compare again the impacts of the redox on Ti, Mn, Fe, and Ni, see Fig. 5 and 6. This is also consistent with the fact that little asymmetry in the PES:s and little effect on activation energies is observed in case of *Q* = +2 (*cf.*Fig. 4), *i.e.* the impact of 3d, 4s and 4p states becomes only marginal in absence of electrons occupying the vacancy.

### III.B. Transition metal attenuated electron hopping

Scale spalling under cyclic high- and low temperature conditions is a common cause for barrier oxide failure of high-temperature alloys. Indeed, the process by which the sought slow-growing well-adhering inner alpha-alumina scale is accessed, comprising initial formation and gradual conversion of a RE-attenuated “junk oxide”, needs thus also to be repeated. This repeated break-down and build-up of oxide scale is the reason why early scale formation is at the heart of high-temperature alloy development. Indeed, also the inward growing oxide may be understood to sustain a transient defect rich oxide “skin” at the receding alloy/oxide interface, and it is by local electrochemical processes that the transient oxide eventually accesses the steady state thermodynamic limit of the growing oxide scale. Repeatedly, these electrochemical processes involve electrons as well as ions transfers. Taking the oxygen vacancies to offer the only mobile ionic species, see previous section, the present section sheds light on ways that transition metal ions impact the electron transfer between said oxygen vacancies having consequences on the stabilization and accumulation of defects, being discussed in chapter III.C.

In what follows, electron transfer between two distant oxygen vacancies in alumina is discussed, and except for the pure alumina case, these are each in immediate proximity to a transition metal ion in the set that includes Al and TM = Sc, Ti, V, Cr, Mn, Fe, Co, Ni. The results are summarized by reaction energy and reaction volume matrices capturing electron transfer associated changes for each of the following reactions

P1.1 B(P_y_^0^;P_x_^+1^) → B(P_y_^+1^;P_x_^0^)

P1.2 B(P_y_^0^;P_x_^+2^) → B(P_y_^+1^;P_x_^+1^)

P1.3 B(P_y_^+1^;P_x_^+1^) → B(P_y_^+2^;P_x_^0^)

P1.4 B(P_y_^+1^;P_x_^+2^) → B(P_y_^+2^;P_x_^+1^)

P1.5 B(P_y_^0^;P_x_^+2^) → B(P_y_^+2^;P_x_^0^)

where B(P_y_^*Q*1^,P_x_^*Q*2^) is used as notation for two spatially separated polarons (a bipolaron) with nominal charges *Q*1, *Q*2 = 0, +1, +2, situated adjacent to the TM dopants y, x = Al, Sc,…, Ni. Thus, five reaction energy & reaction volume matrices (Δ*G*^0^), ten activation energy & activation volume matrices (five for 
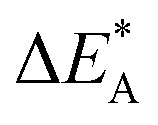
*i.e. E*[*ψ*_A_(*X*_AB_)] − *E*[*ψ*_A_(0)] and five for 
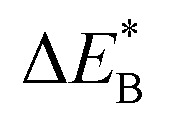
*i.e. E*[*ψ*_B_(*X*_AB_)] − *E*[*ψ*_B_(1)]) are shown in Fig. S4–S6.† Moreover, five maps that summarize the positions of the crossing, *X*_AB_ between *ψ*_A_(*q*) and *ψ*_B_(*q*) along the corresponding reaction coordinate *q* are provided in Fig. S7.†

**Fig. 9:**
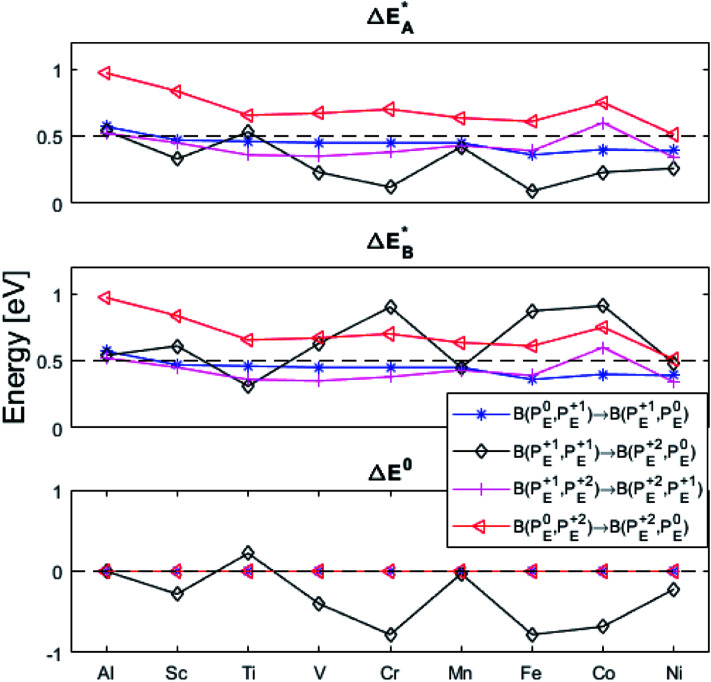
Activation and reaction energies 
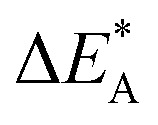
, 
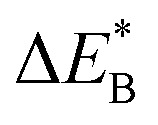
 and Δ*E*^0^ for the diagonal elements in the energy matrices in Fig. S4–S6.†

**Fig. 10:**
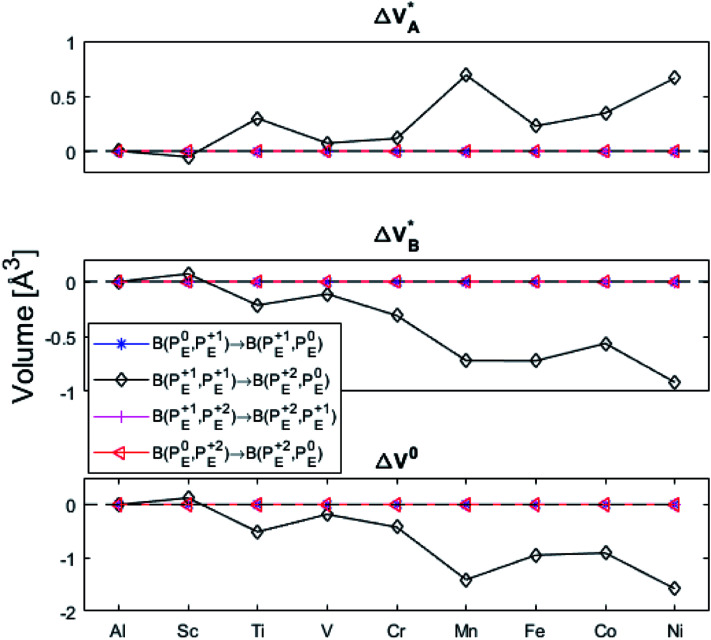
Volume changes for the diagonal elements B(P_*E*_^*Q*1^,P_*E*_^*Q*2^) in the volume matrices in Fig. S4–S6.†

**Fig. 11:**
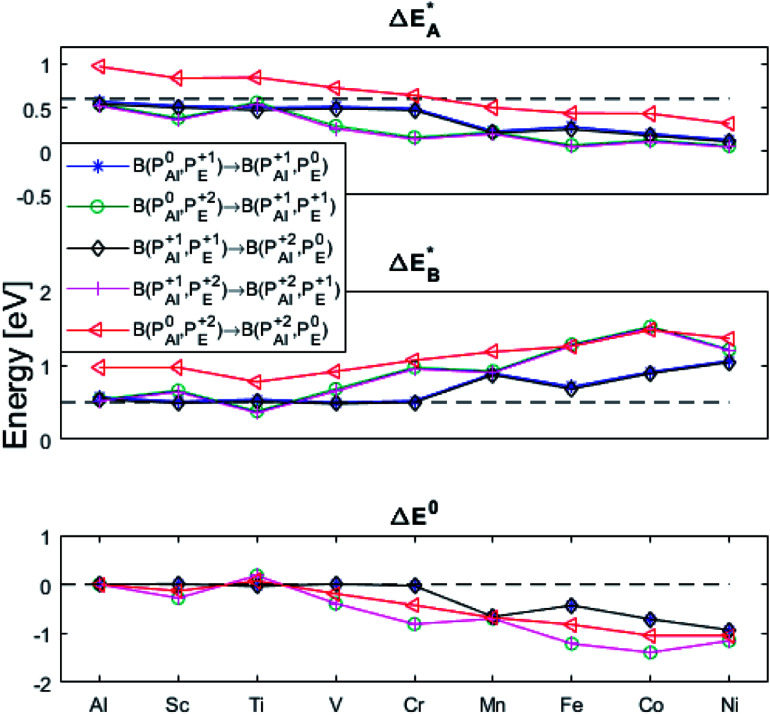
Activation and reaction energies 
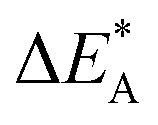
, 
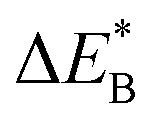
 and Δ*E*^0^ for the last row (y = Al) in the energy matrices in Fig. S4–S6.†

**Fig. 12:**
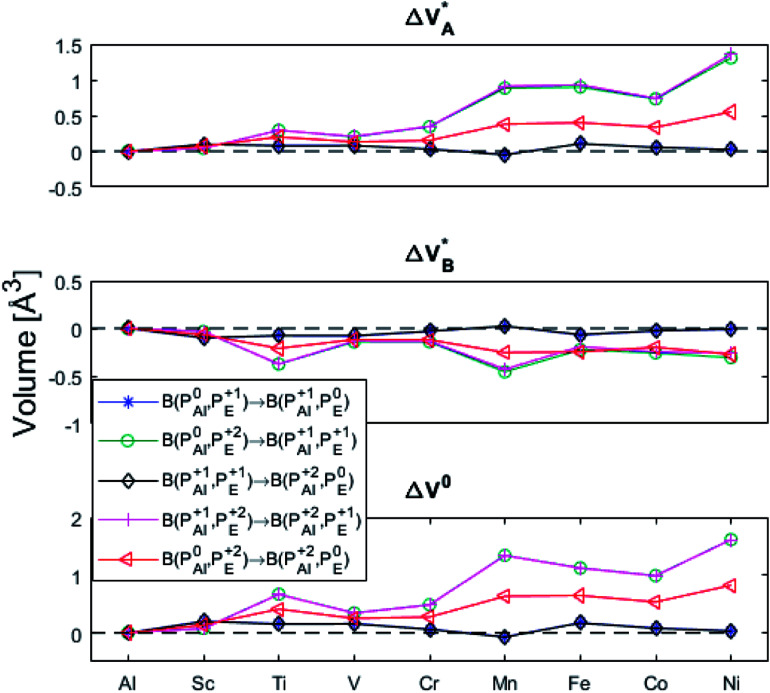
Volume changes for the last row (y = Al) in the volume matrices in Fig. S4–S6.†

Having thus captured all possible pairwise electron transfers between distant oxygen vacancies in the set, it becomes interesting to focus on two specific sub-sets:

(A) Those between equivalent oxygen vacancy compositions, see Fig. 9 and 10 for reaction energy graphs and reaction volume graphs, respectively, the corresponding percolative paths for electron transport becoming relevant in the high TM density regime, *i.e.* in the cases where TM–*V*_O_ couples exist in sufficient density. We may note that for equivalent TM–*V*_O_ couples, y = x = *E i.e.* B(P_*E*_^*Q*1^,P_*E*_^*Q*2^) render the eqn (P1.2) and (P1.3) mutually redundant. The data in Fig. 9 and 10 are identical to those found in the 35 diagonals of the energy, volume, and *q*-matrices in Fig. S4–S7.†

(B) Besides electron transfer between arbitrary TM associated vacancy sites, as summarized in Fig. S4–S7,† indirect transfers *via* an arbitrary number of hops between Al(iii)–*V*_O_ sites are possible. Such an intermediate chain of events is initiated by electron transfer between TM–*V*_O_ and an Al(iii)–*V*_O_ site. It is terminated by opposite electron transfer from an Al(iii)–*V*_O_ to a second transition metal ion associated oxygen vacancy TM*–*V*_O_ site. These initial and final processes comprise the (Al; Al–Ni) columns and (Al–Ni; Al) rows in the matrices presented in Fig. S4–S7.† These are summarized in Fig. 9–12.

First, we make the general observation that the activation energy for single-electron transfer in the pure alumina system is less than 0.5 eV, which is significantly lower than the 2 eV activation energy for diffusion of the most mobile doubly charged oxygen vacancies 
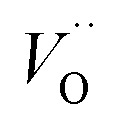
, see Fig. 3, rendering the corresponding redox process to display a catalytic correlation of electron mobility and effective mobility of *V*_O_.

Having demonstrated the pinning of *V*_O_ by TMs in the previous paragraph, it is found here how TM^x^_Al_ ions embedded in an alumina host modify the electron mobility in the *V*_O_ associated impurity band. Indeed, reaction energies related to polaronic trapping of electrons in *V*_O_ sites adjacent to TM^x^_Al_ ions is reported in Fig. 11, while the corresponding structural deformations are shown in Fig. 12. Moreover, it is observed that the trapping of electrons in the oxygen vacancy adjacent to TM increases with atomic number. Correspondingly, the activation energy 
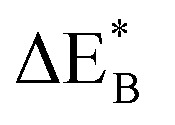
 increases. Note also that the processes involving the reduction of P_*E*_^+1^ to P_*E*_^+0^ (*i.e.* black and blue) are relatively constant up until TM = Cr, beyond which redox properties come into effect for *Q* = 0, +1. Indeed, Mn, Fe, Co, and Ni exhibit significant TM(ii) oxidation state character, compare Fig. 5.

The fact that irrespective of *Q*, the energy difference between P_Al_^*Q*^ → P_Al_^*Q*+1^ is approximately that of P_*E*_^+1^ → P_*E*_^0^ for *E* = {Sc, Ti, V, Cr} is taken to imply that the stability of nominal TM(iii)–*V*_O_(*Q*) can be considered decoupled from TM for Sc to Cr. For *Q* = +2, the bare ionic qualities become decisive, *i.e.* the anisotropic O(−ii)–TM(iii) dative bonding to unoccupied 3d orbitals up until TM = Cr and from TM = Mn and onwards O(−ii) Pauli repulsion towards increasingly occupied 3d orbitals render the nominal O(−ii)–TM(iii) system increasingly destabilized. The latter effect in turn, is the reason for the increased stability of nominal TM(iii)–
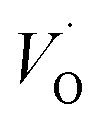
 and TM(iii)–*V*_O_ in the redox processes involving P_*E*_^+2^ → P_*E*_^+1^ and P_*E*_^+1^ → P_*E*_^0^, respectively. Still, the degree to which said two reduction processes become approximately independent of whether *Q* = 0 or +1 in the corresponding vacancy oxidation processes P_Al_^*Q*^ → P_Al_^*Q*+1^ (*Q* = 0 for purple and green; *Q* = +1: blue and black) is notable. This is an effect of the introduced background charge compensating for the charged super cells, see Section S2† for a more detailed discussion.

**Fig. 13:**
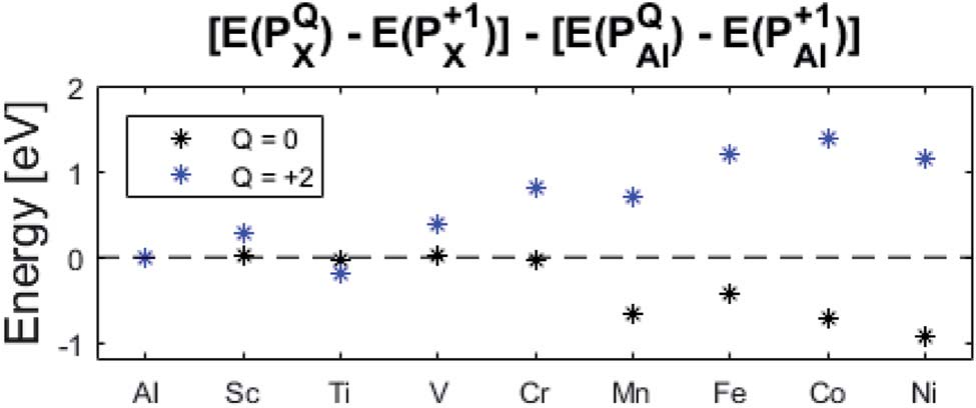
Relative energies between *Q* = 0, +1, and +2 structures, relative to *Q* = +1 (dotted line). It is noted that the X = Mn values happen to be centered around 0 eV which results in *q* = 0.5 while Fe, Co, and Ni have *q* > 0.5. Ti (*Q* = +2) has the lowest relative energy of its' charge states, due to allowed Ti(iv) state.

The confluence of reaction energies for the different redox processes in case of TM = Mn is unexpected, and particularly so since similarities to Fe, Co, and Ni are expected from the study on the mobility of oxygen vacancy, as also supported by the volume changes in Fig. 12. An aspect of this anomaly is addressed in Fig. 13 where the energy differences of TM–*V*_O_ (*Q* = 0) and TM–
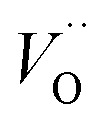
 (*Q* = +2) relative to TM–
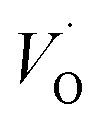
 (*Q* = +1) and offsetted by the corresponding values for Al are displayed. It is observed that the (*Q* = +2) values follow a quasi-regular pattern related to the gradual filling of the 3d sub-shell, this while the (*Q* = 0) values remain zero until TM = Mn where it deviates due to redox properties. Accidentally, the stabilities of Mn–*V*_O_ (*Q* = 0) and Mn–
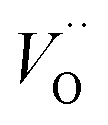
 (*Q* = +2) come out symmetric around 0 eV, and as a consequence the reaction coordinate of *q* = 0.5 is obtained. It is also reassuring that nominal Ti(iii)–
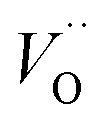
 (*Q* = +2) comes out more stable than nominal Ti(iii)–
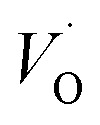
, see Fig. 13 again. The reason for this effect is repeatedly that the *Q* = +2 moiety undergoes an internal redox process to form Ti(iv)–
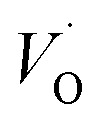
 (*Q* = +2).

Moreover, it is gratifying to note the extent to which the obtained reaction energies map on the corresponding volume changes, see Fig. S6† again. Indeed, when deviations occur, these are ascribed to a mixed TM oxidation states in the redox the processes. Also handy is how the trapping effect can be extracted from the position of the transition state along the redox reaction coordinate X_AB_, see Fig. S7.† Thus, X_AB_ < 0.5 (X_AB_ > 0.5) tell of reactant-like (product-like) redox processes for the nominally TM(iii) associated oxygen vacancies becoming reduced coupled to the oxygen vacancies in alumina being oxidized.

### III.C. Novel perspective on “Third Element Effect”

Computed trends, from 1st principles, for 3d transition metal guest ions attracting oxygen vacancies in the corundum structure of α-alumina, dependency on charge of the vacancy, corresponding impact on the activation energies for oxygen vacancy diffusion as well as the impact of redox properties at the transition state were presented in paragraph III.A. Moreover, systematic attenuations of TM ions adjacent to the oxygen vacancies that offer impurity states for electron hopping conductivity were elucidated in paragraph III.B, including charge dependences of the corresponding (bi-)polaronic effects.

In as much as these transport properties are relevant for the conversion of *e.g.* a transient oxide into one that obeys local thermodynamic conditions, here, we put the findings in the context of the so-called 3rd element effect. Thus, beneficial oxide scaling properties may be achieved by adding a third element to a binary alloy. The most well studied systems are the iron base alumina forming alloys. In these, it is common to add Cr as the third element, its oxides displaying intermediate stability.^7,14^ Thus, internal oxidation of Al is avoided as external oxidation of the 3rd element is achieved. Additional Cr is introduced in order to suppress formation of the iron oxide component in the transient external oxide that precedes the thermodynamic scale formation. Because Al_2_O_3_ and Cr_2_O_3_ are completely miscible at high temperatures, the total concentration N_Cr_ + N_Al_ is increased by the presence of Cr at the oxide scale interface, thus promoting formation of corundum type (Al,Cr)_2_O_3_.

Repeatedly, a necessary condition for achieving the 3rd element effect is that its oxides display intermediate stability, *e.g.* chromia is more stable than the iron oxides but less stable than alumina. From the observations offered in the present study, it becomes tempting to reflect on the possible uniqueness of Cr as third element accompanying Fe and Al. Thus, for the Fe–TM–Al system, the possible third elements are those elements in periodic table which are between Fe and Al in the Ellingham diagram. Here, Sc, Ti, V, Cr, and Mn are possible candidates. Indeed, it may be argued that a good candidate for 3rd element should be one that is miscible in alumina and a stronger oxygen-getter than iron. The present study does not address miscibility, yet, the stability of the TM(iii) oxidation state may be employed as an indicator. Here, Mn and Ti exclude themselves as they maintain oxidation states in the alumina matrix that differs from the +III of Al in alumina. Moreover, similar affinities to oxygen vacancies as Fe suggests these ions to segregate to the interfaces of alumina grains. Sc, although maintaining a +III oxidation state shows very similar vacancy mobility and electronic transport properties as Al, and should not facilitate the transformation of the metastable initial oxide. Thus, we arrive at the two remaining candidates, Cr and V, who both maintain a +III oxidation state in the alumina matrix, suggesting facile initial dissolution in the transient oxide. Also, in as much as both oxygen vacancy mobility and electron transfer become facilitated by the substitutional doping of Cr and V at the Al(iii) site, then consequently, so is also the transformation from metastable scale into the sought oxide reflecting the local thermodynamic conditions, that is α-alumina. Indeed, the differences between the two are small, see Tables 1 and 2 for transport properties emerging from Fig. 11 and 12. It is concluded that V and Cr come out fairly equal as candidate 3rd elements in the Fe–TM–Al system, and that properties other than those addressed here become decisive for discriminating between the two.

**Table 1:** Activation energies for oxygen vacancy diffusion (eV), ΔE* is Δ*E*(TS-Start). Δ*E*_R_ (eV) tells of the APES for *V*^x^_O_, 
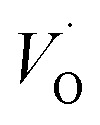
, and 
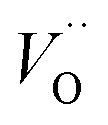
 respectively, in case of TM = V, Cr

	ΔE*, [*V*^x^_O_], Δ*E*_R_		ΔE*, 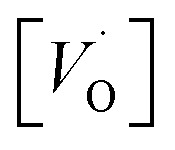 , Δ*E*_R_		ΔE*, 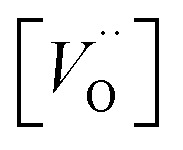 , Δ*E*_R_	
V	3.7	0.6	2.3	0.6	1.6	0.2
Cr	3.7	0.8	2.4	0.7	1.6	−0.1

**Table 2:** Electron transfer activation energies 
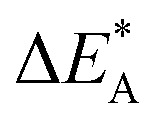
 for the five processes detailed in Section III.B for TM = V, Cr. Data for coupling of two equivalent TM associated vacancies is extracted from Fig. 9. Data for coupling of two inequivalent oxygen vacancies, one TM associated and one non-TM associated, is taken from Fig. 11. Energies are in eV

	(P1.1)	(P1.2)	(P1.3)	(P1.4)	(P1.5)
V⇔V	0.45	0.23	0.63	0.35	0.67
Cr⇔Cr	0.45	0.12	0.90	0.38	0.70
V⇔Al	0.51	0.29	0.49	0.26	0.73
Cr⇔Al	0.49	0.16	0.47	0.14	0.64

The overall purpose of the TM additions becomes to enhance the initial stability of the transient “junk oxide” as well as to improve on its adhesion to the alloy support. Thereby, spalling is avoided during early stages of scale growth. Indeed, increased formation of junk oxide is not necessarily negative as it enhances the Al activity, which facilitates the formation of a protective alumina scale. Accelerated transformation to access the local equilibrium composition is assisted by “the 3rd element effect”. In *e.g.* FeCrAl its effect is to render the resulting alumina based (Al,Cr)_2_O_3_ corundum scale fully oxidized. Oxygen vacancies are either accommodated in an outer non-protective iron oxide or accumulate as voids at the alloy/oxide interface, *i.e.* become associated to the most noble metal, here iron. The latter impairs scale/alloy adhesion. A remedy utilizes the reactive element effect, *i.e.* minor RE additions to the alloy inhibit early grains coarsening by RE ions decorating alumina grain boundaries.^8^ Thus, parabolic scale growth is maintained for a longer period, prior to grains coarsening sets in which renders the scale growth kinetics cubic.^48^

## IV. Summary and conclusion

Conversion of the initial rapidly formed transient oxide into an oxide scale that obeys local thermodynamic equilibrium conditions on alumina formers is a complex process. Following incomplete initial oxidation, secondary oxidation is common at later stages as manifested by growth of both columnar and equiaxial alumina grains.

The present study is dedicated to the unravelling of electron and ion transport processes essential for the 3rd element effect. It is gratifying to note the extent to which empirical studies provide experimental evidence that capture essential technological implications, already formulating the said 3rd element effect as well as the “reactive element effects”.^2,7,20^ Here, we provide a mechanistic perspective that bridges over to the atomic length scale.

In the present study, oxygen deficiency in an α-alumina scale was modelled by the introduction of oxygen vacancies. Moreover, in as much as alumina forming alloys include *e.g.* FeCrAl, FeNiCrAl, NiCrAl, CoCrAl, it became essential to investigate the impact of guest ions (TM = Sc, Ti, V, Cr, Mn, Fe, Co, Ni) on oxygen vacancy mobility as well as electron transfer.

Emerging from this study is the crucial coupling between two processes that control the oxide growth kinetics. Thus, oxygen vacancy diffusion was shown to be highly dependent on the charge of the oxygen vacancy, *i.e.* whether *V*_O_, 
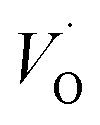
, or 
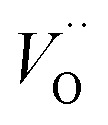
. Electron transfer among redox active oxygen vacancies in defect rich α-Al_2_O_3_ was proposed to sustain electro-catalytic oxygen vacancy mobility. Support for this notion was provided by the computed activation energies for electron hopping between oxygen vacancies that come out significantly lower than those for oxygen vacancy diffusion, *i.e. E*_A_ ∼ 0.5 eV for the electron hopping and 
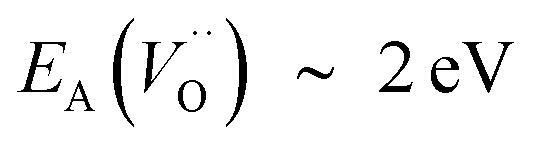
, 
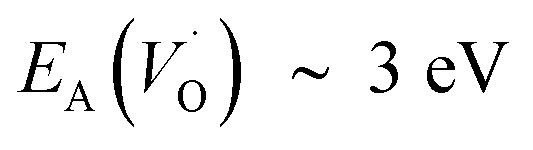
 and *E*_A_(*V*_O_) ∼ 5 eV. A correlated oxidation process in pure alumina merges in that the non-rate limiting electron transfer between oxygen vacancies render the resulting mobility of the oxygen vacancies enhanced.

Oxygen vacancies become pinned by TM in the alumina scale, as seen in the asymmetry in the potential energy surface reflecting the corresponding affinity as compared to the alumina host, is 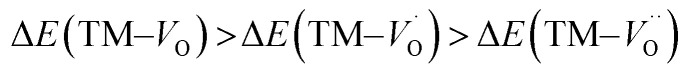. The TM assisted stabilization of an adjacent oxygen vacancy is owing to the 4s and 4p states that serve to stabilize the electronic state associated with the vacancy when occupied by electron(s). The activation energy for compound formation is also affected by the TM. But rather than the 4s and 4p states, in this case it is the 3d shell that is decisive. In as much as the activation energy is controlled by the charge of the vacancy, the mixed oxidation state 3d^*n*±1^ of TM(iii) at the transition state decides the resulting height of the barrier for diffusion of *V*_O_ and 
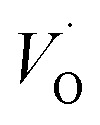
. Again, the higher the charge, the lower the activation energy. Interestingly, in case of TM = Ti, the opposite effect is observed since Ti(iii)–
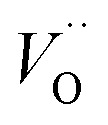
 is converted into Ti(iv)–
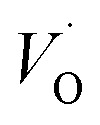
 rendering the apparent activation energy for TM–
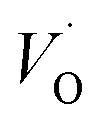
 and TM–
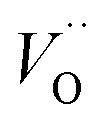
 similar in this case.

Two ways how the TM guest ion impacts the electron mobility in the oxide scale were described. Firstly, charge dependent polaronic effects on the oxygen vacancies owing to the differential relaxation of the local alumina lattice impedes the electron mobility. Secondly, reduced *V*_O_ sites adjacent to TM becomes increasingly preferred, *e.g.* for TM = Mn, Fe, Co, Ni, rendering the defect structure less mobile. This line was taken one step further by quantifying all pairwise competitions for electrons in donor–acceptor set-ups composed of two of the TM associated oxygen vacancies considered. Indeed, the nobler TM is found to prefer the reduced oxygen vacancy for all five different electron transfer processes. For a ternary alloy system, *e.g.* iron base alloy where iron constitutes one of the guest elements in the alumina host, the condition on any 3rd element to display intermediate affinity to an uncharged oxygen vacancy points at vanadium and chromium as two viable candidates to display the resulting so-called 3rd element effect.

## Conflicts of interest

There are no conflicts to declare.

## Supplementary Material

RA-008-C8RA08195F-s001
