# Supplementary material for: Transition metal attenuated mechanism for protective alumina formation from first principles
Source: RSC Adv. 2018 Dec 11;8(72):41255–69. doi: 10.1039/c8ra08195f (PMC9091954; doi:10.1039/c8ra08195f)
Supplement: RA-008-C8RA08195F-s001 [file RA-008-C8RA08195F-s001.pdf]

Electronic Supplementary Material (ESI) for RSC Advances.
This journal is © The Royal Society of Chemistry 2018

Electronic Supplementary Information

Transition Metal Attenuated Mechanism for Protective Alumina Formation from First Principles

Vedad Babic,*[a] Christine Geers[a] and Itai Panas[a]

Table S1. Total energies [eV] of $Al_{47}O_{71}TM$ super cells where the oxygen vacancy (V_O) position is varied w.r.t. TM-V_O distance. All energies relative to vacancy at 5.1 Å. See Figure S1.

Distance [Å]	**2.0 [Start]**	**3.5**	**3.9**	**4.4 [End]**	**5.1**
**Sc**	-0.342	-0.003	-0.055	-0.086	0
**Co**	-1.71	0.000	-0.009	-0.122	0
**Ti**	-0.653	0.006	0.026	-0.035	0

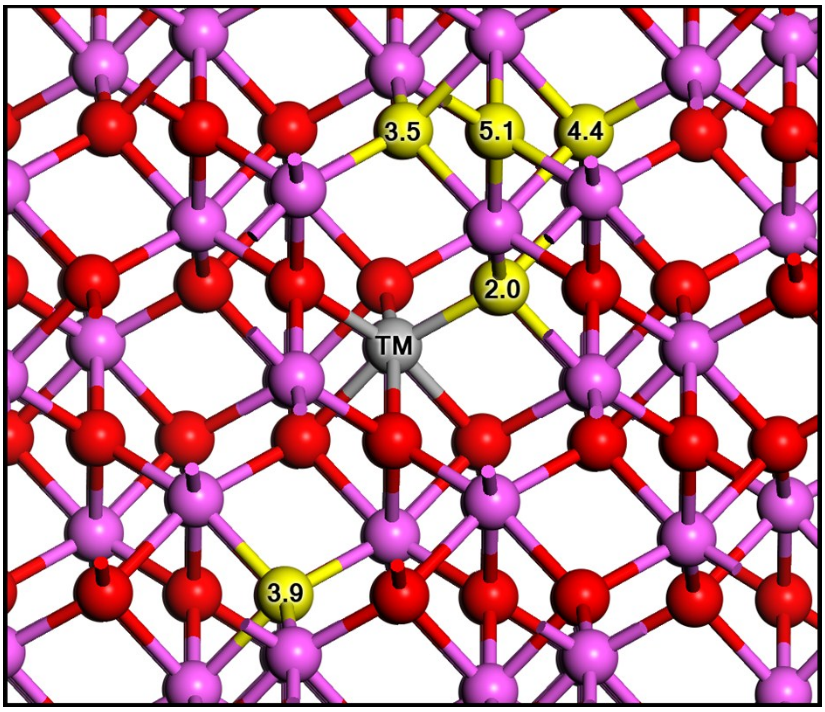

Figure S1. The oxygen ions which are deleted to form vacancies in Table S1. Numbers denote the TM-O distance in Ångström.

Table S2. ΔE^*_A, ΔE^*_B, and APES, Q = 0, for various TM's with specified spin state in brackets. In case multiple computed spin states, the ground state is specified w.r.t. "End" geometry and is **bold.** All results in eV.

TM (Spin)	**Al (0)**	**Sc (0)**	**Ti (1)**	**V (2)**	**Cr (3)**	**Mn (4)**
ΔE^*_A	**4.6**	**4.4**	**4.1**	**3.7**	**3.7**	**3.3**
ΔE^*_B	**4.6**	**4.1**	**3.5**	**3.1**	**2.9**	**1.9**
$APES(0)$	**0.0**	**0.3**	**0.6**	**0.6**	**0.8**	**1.4**
Fe (1)	**Fe (3)**	Fe (5)	**Co (0)**	Co (2)	**Ni (1)**	**Ga (0)**
3.4	**3.1**	3.2	**3.4**	2.8	**2.9**	**4.5**
2.2	**1.7**	1.9	**1.8**	1.3	**1.4**	**3.0**
1.2	**1.4**	1.3	**1.6**	1.6	**1.5**	**1.5**

Table S3. ΔE^*_A, ΔE^*_B, and APES, Q = +1, for various TM's with specified spin state in brackets. In case multiple computed spin states, the ground state is specified w.r.t. "End" geometry and is **bold.** All results in eV.

TM (Spin)	**Al (1)**	**Sc (1)**	Ti (0)	**Ti (2)**	V (1)	**V (3)**
ΔE^*_A	**3.1**	**2.9**	3.4	**2.6**	2.8	**2.3**
ΔE^*_B	**3.1**	**2.6**	3.1	**2.1**	2.4	**1.7**
$APES(+1)$	**0.0**	**0.3**	0.3	**0.5**	0.4	**0.6**
	**Cr (4)**	Mn (3)	**Mn (5)**	**Fe (4)**	**Co (1)**	**Ni (2)**
	**2.4**	2.5	**1.8**	**2.0**	**2.0**	**1.8**
	**1.6**	1.3	**0.8**	**0.9**	**0.8**	**1.0**
	**0.7**	1.3	**1.0**	**1.1**	**1.1**	**0.8**

Table S4. ΔE^*_A, ΔE^*_B, and APES, Q = +2, for various TM's with specified spin state in brackets. In case multiple computed spin states, the ground state is specified w.r.t. "End" geometry and is **bold.** All results in eV.

TM (Spin)	**Al (0)**	**Sc (0)**	**Ti (1)**	**V (2)**	**Cr (3)**	**Ni (1)**
ΔE^*_A	**1.7**	**1.4**	**1.9**	**1.6**	**1.6**	**1.8**
ΔE^*_B	**1.7**	**1.5**	**1.9**	**1.4**	**1.7**	**1.1**
$APES(+2)$	**0.0**	**-0.1**	**0.1**	**0.2**	**-0.1**	**0.7**
Mn (0)	Mn (2)	**Mn (4)**	Fe (1)	**Fe (3)**	**Co (0)**	Co (2)
-	-	**1.7**	1.7	**1.8**	**1.7**	1.7
-	-	**1.0**	1.6	**1.1**	**1.8**	1.1
0.0	0.1	**0.7**	0.1	**0.7**	**-0.1**	0.7

S1 – Asymmetry in the potential energy surface (APES)

Signature of the APES is sought in the changes in oxygen bond lengths towards the five-coordinated TM ion where the sixth coordination is towards the oxygen vacancy, see Figure S2. It is observed how

the O_5 becomes a sensitive probe of the vacancy charge. Successively, for Q = 0, +1, +2 the TM-O_5 bond goes from being significantly longer than the average TM-O bond to becoming increasingly similar to the average for Q = +2. Repulsion towards O_5 anion is taken to reflect an electron rich TM-V_O moiety or Pauli repulsion towards the TM. In case of Q = 0, significant TM-O_5 elongation as compared to Al-O_5 is observed. This suggests greater attraction between oxygen vacancy and TMs than to Al. The effect prevails to some extent in case of Q = +1, cf. right panel in Figure S2 (centre graph) and APES in Figure 3A & 3B. For Q = +2, the small difference between TM-O_5 and Al-O_5 bond distances is taken to reflect bare Pauli repulsion between O_5 and the 3d shells in case of TM, see Figure S2 right panel again (bottom graph), including spin dependent anomalies for TM = Mn, Fe, Co, Ni. The Q = +2 APES apparently reflects spin dependent geometrical relaxations utilizing the vacancy, thus favouring the Start structure. Indeed, on increasing the occupation of the 3d shell, high-spin states tend to cause more Pauli repulsion toward the O_5 owing to ligand field effects, see ref. [S1]. In case of Mn, the connection between O_5-TM bond distance and spin density along the O_5-TM-V_O axis comes out clearly, see Figure S3. This effect in turn is taken to disfavour a vicinal O_6 residing in the V_O, when comparing to the O_5-Al-O_6 structure, and thus cause APES for Q = +2.

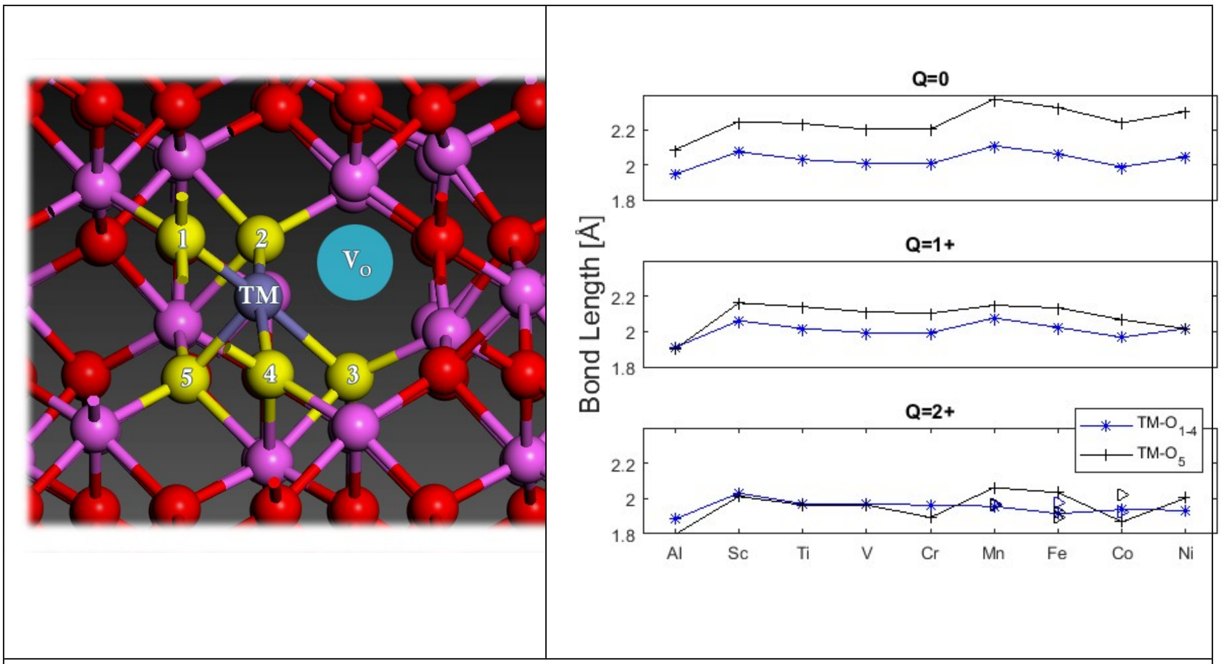

FIGURE S2. Left: The TM-ion with five nearest-neighbor oxygen ions in the start structure. Note that O_1-O_4 are lying in the plane perpendicular to the O_5-TM-V_O axis. Right: Bond lengths are plotted

for O_1-O_4 (mean value) and O_5 for Q = 0 (top); Q = +1 (centre); Q = +2 (bottom). Triangles denote exited spin states. Gradual quasi-monotonic shrinking of TM-O_5 distances with increased charge is shown (top-centre-bottom), while negligible effects of charge on average TM-(O_1-O_4) is found. Bond distance variations for (Q = +2) TM-O_5 reflect those in Figure 4c. For impact of spin-state on bond distance modulations, see text.

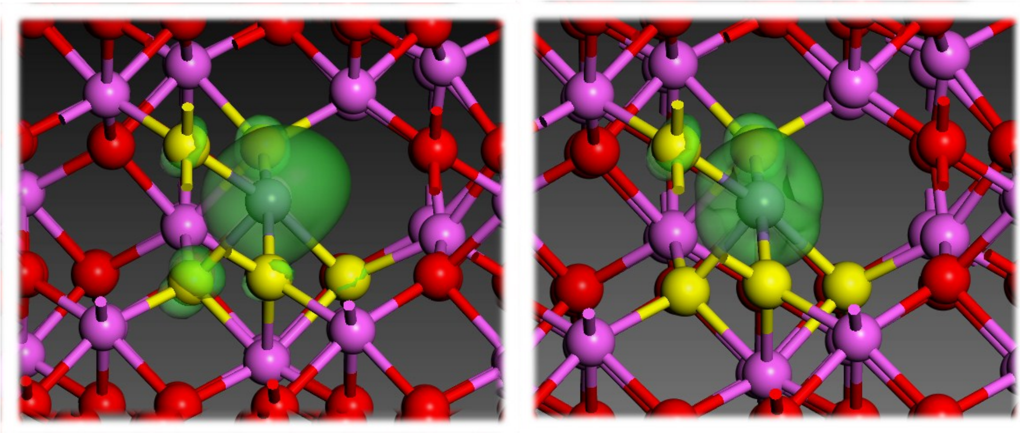

FIGURE S3. *Left:* The ground state spin density of nominal Mn(III)-$V_O^{\bullet\bullet}$ (Q = +2). *Right:* Excited state of the system. Note the spin density on O_5 which disappears in case of the excited state.

Additionally, the fact that Q = +2 displays very little APES suggest that the 3d shell to some extent takes a spectator role. Thus, the dramatic 3d bands shifting relative to the Fermi energy with increasing atom number is taken to exclusively signify shrinking of the TM ions due to the increased nuclear charge, see Figure 2 again. That APES results from 3d binding to the vacancy is contradicted by the absence of spin dependence in the APES of nominal Fe(III)-V_O ($Q = 0$), i.e. not differentiating between high-spin and low-spin stabilities, see Table S2.

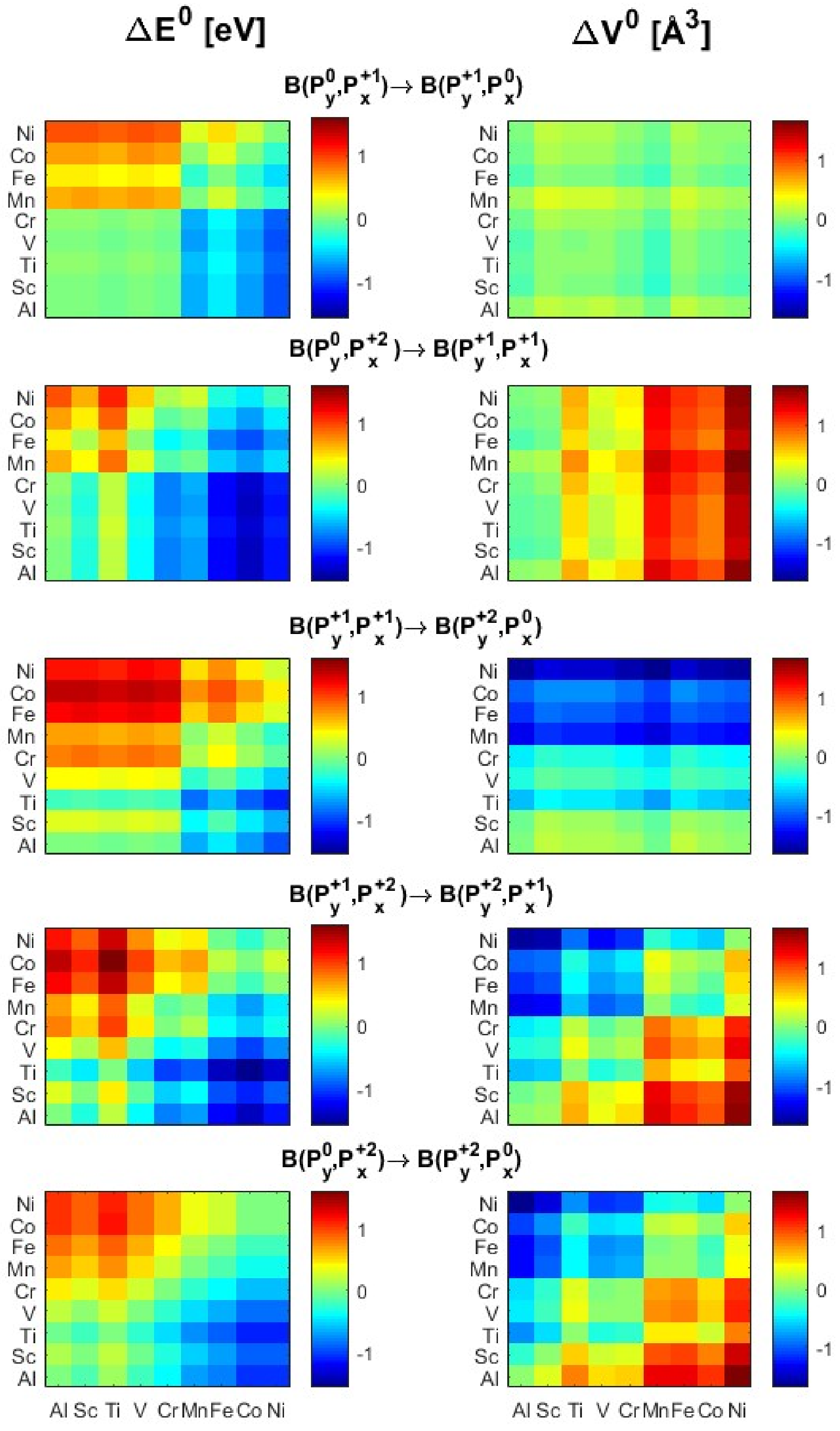

FIGURE S4. (left) Reaction energy matrices for ΔE^0 and (right) corresponding reaction volume matrices for equations P1.1-P1.5.

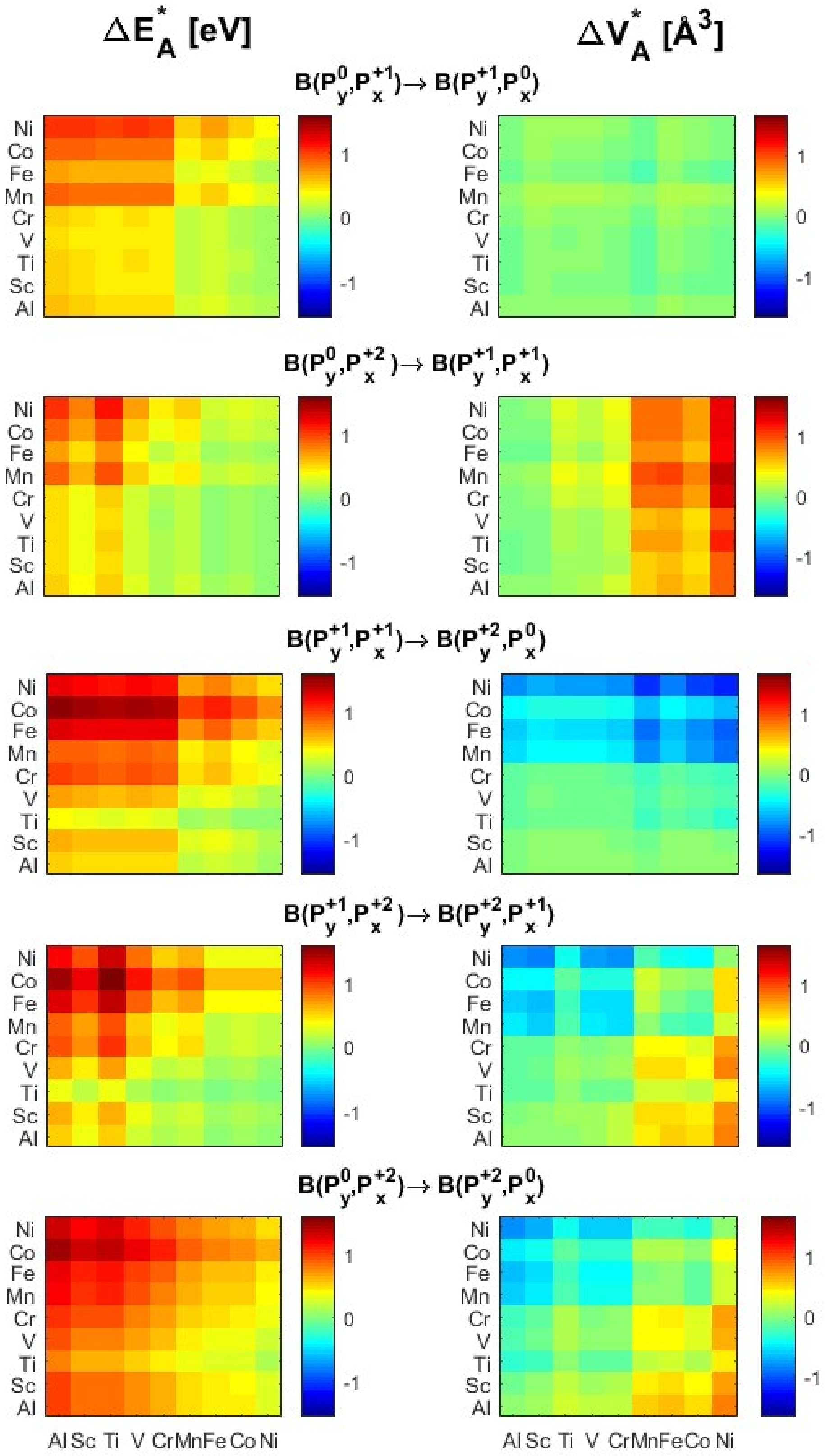

FIGURE S5. (left) Reaction energy matrices for ΔE_A^* and (right) corresponding reaction volume matrices for equations P1.1-P1.5.

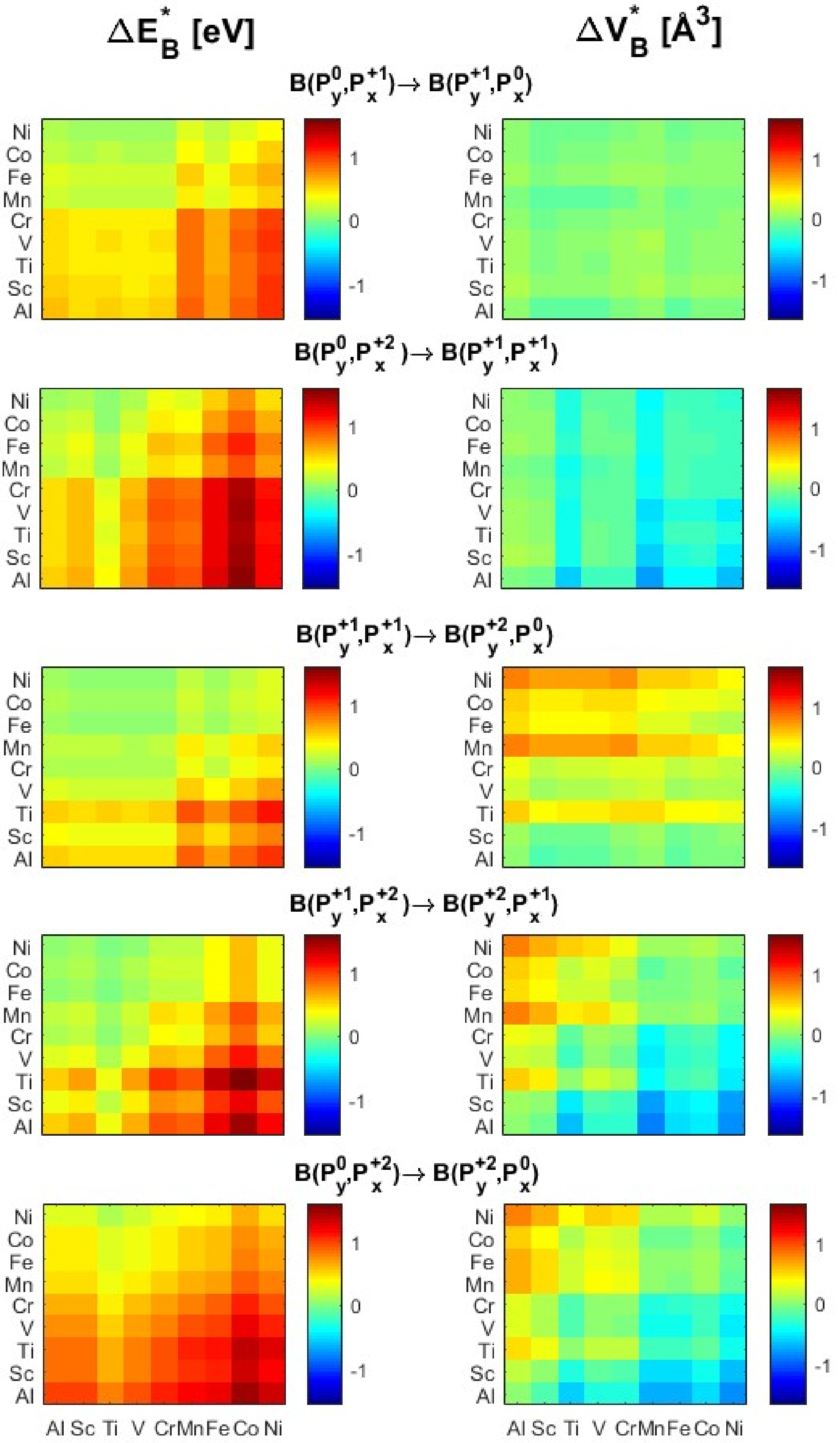

FIGURE S6. (left) Reaction energy maps for ΔE^*_B and (right) corresponding reaction volume maps for equations P1.1-P1.5.

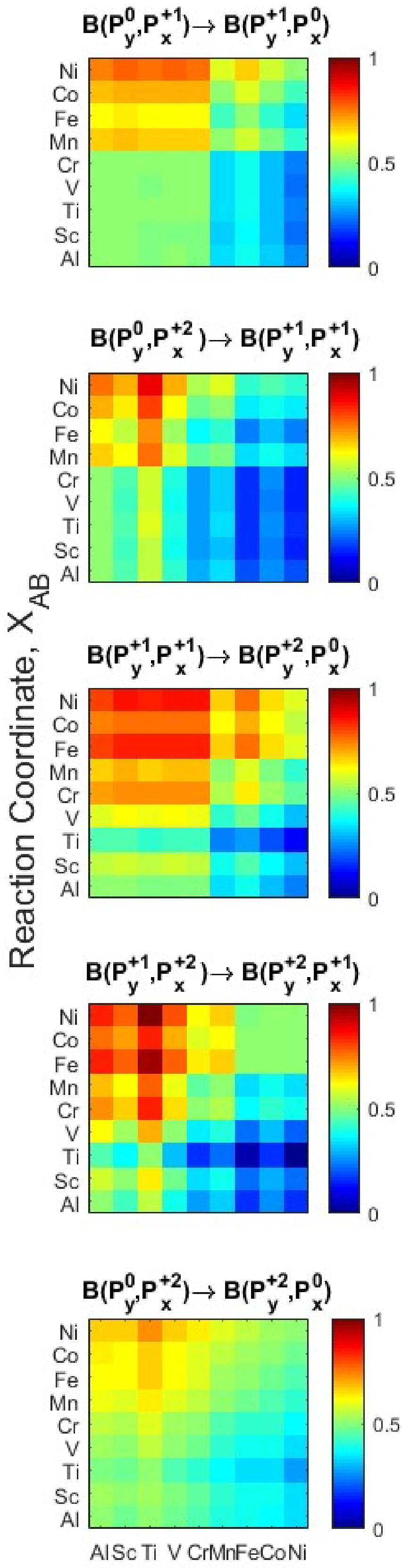

FIGURE S7. Reaction coordinate X_{AB}, cf. Figure 2 of the crossing between $\Psi_A(q)$ and $\Psi_B(q)$ for processes P1.1-P1.5.

S2 – Asymmetric polaron transfer processes P1.2 and P1.3

It should be born in mind throughout the present paragraph that it is differential effects regarding electron trapping among TM associated oxygen vacancies which are sought, i.e. preference for the reduction of the oxygen vacancy site adjacent to TM(III) on expense of an oxygen vacancy in alumina which becomes oxidized. While charging in the electron transfer reactions generally cancel, this does not apply in case of the asymmetric reactions

$$B(P_{Al}^{0},P_{X}^{+2})\rightarrow B(P_{Al}^{+1},P_{X}^{+1}) \qquad P1.2$$

$$B(P_{Al}^{+1},P_{X}^{+1})\rightarrow B(P_{Al}^{+2},P_{X}^{0}) \qquad P1.3$$

Thus, numerical similarities with the corresponding reactions

$$B(P_{Al}^{+1},P_{X}^{+2})\rightarrow B(P_{Al}^{+2},P_{X}^{+1}) \qquad P1.4$$

$$B(P_{Al}^{0},P_{X}^{+1})\rightarrow B(P_{Al}^{1+},P_{X}^{0}) \qquad P1.1$$

become artefacts of the background charge density that renders the charged supercells screened. The connection between the two pairs becomes obvious upon appropriately offsetting the charging effect by symmetrizing the asymmetric equations analogous to Figure 13, see acute brackets below,

$$B(P_{Al}^{0},P_{E}^{+2}) + [B(P_{Al}^{+1},P_{Al}^{+1})]\rightarrow B(P_{Al}^{+1},P_{E}^{+1}) + [B(P_{Al}^{+2},P_{Al}^{0})]$$

$$\Leftrightarrow$$

$$B(P_{Al}^{+1},P_{E}^{+2})\rightarrow B(P_{Al}^{+2},P_{E}^{+1})$$

and

$$B(P_{Al}^{+1},P_{E}^{+1}) + [B(P_{Al}^{+2},P_{Al}^{0})]\rightarrow B(P_{Al}^{+2},P_{E}^{0}) + [B(P_{Al}^{+1},P_{Al}^{+1})]$$

$$\Leftrightarrow$$

$$B(P_{Al}^{0},P_{E}^{+1})\rightarrow B(P_{Al}^{+1},P_{E}^{0})$$

References

S1 M. Lindgren and I. Panas, *RSC Adv.*, 2013, 3, 21613-21619.
